# A retrospective study on the short-term efficacy of injection and manipulation in primary frozen shoulder

**DOI:** 10.1097/MD.0000000000042310

**Published:** 2025-05-16

**Authors:** Cheng-Wei Kang, Hong-Yun Wo, Li-Xue Wu, Zhao-Kui Yan

**Affiliations:** a Department of Orthopaedics, West China School of Public Health and West China Fourth Hospital, Sichuan University, Chengdu, Sichuan, China; b Department of Geriatric Care and Palliative Medicine, West China School of Public Health and West China Fourth Hospital, Sichuan University, Chengdu, Sichuan, China; c Department of Pathology, West China School of Public Health and West China Fourth Hospital, Sichuan University, Chengdu, Sichuan, China.

**Keywords:** Constant–Murley score, frozen shoulder, intra-articular injection, manipulation, McGill Pain Scale

## Abstract

There is no consensus on the optimal treatment approach for frozen shoulder. This study aimed to evaluate the short-term clinical efficacy of intra-articular injections combined with manipulative therapy, intra-articular injections alone, and local steroid pain point injection for the treatment of primary frozen shoulder. A retrospective study was conducted on 202 patients with frozen shoulder who visited the Department of Orthopedics from January 2020 to April 2022. This study followed the Consolidated Standards of Reporting Trials guidelines for reporting retrospective studies. The patients underwent 3 treatment modalities: intra-articular injection combined with manipulation (lidocaine 100 mg, betamethasone 5 mg, and saline 100 mL), intra-articular injection alone (lidocaine 100 mg, betamethasone 5 mg, and saline 100 mL), and local steroid pain point injection (lidocaine 100 mg, betamethasone 5 mg, and saline 5 mL). The patient’s pain and functional improvement were assessed using the Simplified McGill Pain Scale and the Constant–Murley score before treatment, and at 6 and 12 months after treatment. A systematic review of the literature on hydrodilatation with corticosteroids for the treatment of frozen shoulders was also conducted. There were no differences in baseline indicators among the 3 treatment groups. At the 6-month and 12-month follow-up visits, the McGill pain scores of the intra-articular injection combined with manipulation group were significantly lower than those of the other groups (*P* < .001), while the Constant–Murley scores were significantly higher (*P* < .001). After 12 months of follow-up, the total effective rate was 94.6% for the intra-articular injection combined with manipulation group, 90.9% for the intra-articular injection group, and 87.5% for the local steroid pain point injection group. Intra-articular injection combined with manipulative release can rapidly alleviate pain symptoms and improve joint function in patients with frozen shoulder. This procedure is simple, safe, and suitable for use in medical institutions of all levels.

## 1. Introduction

Frozen shoulder (FS), also known as adhesive capsulitis, is a common shoulder disorder affecting approximately 3% to 5% of the general population.^[1,2]^ FS is characterized by shoulder pain and progressive limitation of activities, with 3 distinct phases: the painful phase (2–9 months), the stiff phase (4–12 months), and the recovery phase (5–24 months).^[3]^ The pathological changes in patients with FS often include intracapsular stiffness in the glenohumeral joint capsule.^[3]^ The pathological changes in frozen shoulder involve intracapsular stiffness in the glenohumeral joint capsule, including synovial inflammation, synovial edema, congestion, hypertrophy of the villi with fibrin exudation, and contracture of the joint capsule and ligaments.^[4]^ Multiple bursae and tendon stops, such as the subacromial or subdeltoid bursa, subscapularis bursa, and brachioradialis bursa, as well as the long head tendon of the biceps and the supraspinatus, subscapularis, and rostro-humeral ligaments, are often involved. (see Figure S1, Supplemental Digital Content, https://links.lww.com/MD/O777 for a typical frozen shoulder MRI presentation).

Although frozen shoulder is a self-limiting condition, functional recovery of the shoulder is often incomplete.^[5]^ Currently, there is no national or international consensus on the optimal treatment of FS; however, current treatments include medication, suprascapular nerve block, arthroscopic release, exercise therapy, and physical rehabilitation.^[5,6]^ Local steroid injections are commonly used to suppress synovial inflammation and provide pain relief in the early stages of the disease,^[7]^ and intra-articular injections have also gained popularity in clinical practice. Ultrasound-guided injections are considered more accurate but are more costly and time-consuming than non-ultrasound-guided injections. Manipulation under anesthesia can improve function, alleviate pain, and enhance passive ROM,^[8,9]^ the disadvantage is that patients require general anesthesia in the operating room, which entails anesthesia-related risks and higher costs. Therefore, this retrospective study aimed to compare the short-term clinical efficacy of local steroid pain point injection, intra-articular injection alone, and intra-articular injection combined with manipulative release for the treatment of primary frozen shoulder. Additionally, a systematic review of the literature over the past 20 years on the use of corticosteroid hydrodilatation injections for frozen shoulder was conducted.

## 2. Methods

### 2.1. General information

This retrospective study was conducted at the Department of Orthopedics of our institution. This study followed the Consolidated Standards of Reporting Trials guidelines for reporting retrospective studies. The study was approved by the Ethics Committee of West China School of Public Health/West China Fourth Hospital of Sichuan University (HXSY-EC-2022048). Informed consent was obtained from all participants before treatment.

According to previous literature reports,^[10]^ the sample size was determined using PASS11 software, considering a Type I error of 0.05 (bilateral) and a certainty of 0.80, with an expected failure rate of 20%. At least 30 participants were included in each group. By conducting a comprehensive search of both outpatient and inpatient cases within our hospital, a total of 202 patients diagnosed with primary frozen shoulder, who sought treatment at our orthopedic department between January 2020 and April 2022, were included in this study.

### 2.2. Inclusion criteria

① Patients were included if they met the diagnostic criteria for frozen shoulder^[5]^; ② shoulder joint pain with activity function limitation (at least 2 directions passive activity limitation, especially abduction, external rotation and posterior extension and internal rotation); ③ shoulder joint Simplified McGill Pain Scale (SF-MPQ) pain score of ≥ 30 and a Constant–Murley function score (CMS) of ≤ 50; ④ X-ray imaging of the shoulder joint did not reveal any clear evidence of bony abnormalities.

### 2.3. Exclusion criteria

① Patients with full-layer rotator cuff tear (patients with partial rotator cuff tears with frozen shoulder as the main clinical manifestation were still included in the study); ② acromioclavicular impingement; ③ metastatic shoulder bone tumor; ④ acute shoulder trauma; ⑤ combination of other diseases affecting the shoulder joint, such as rheumatoid arthritis, metabolic bone disease, calcific tendonitis of the supraspinatus muscle, cervical spine disease, brachial plexus nerve involvement disease, etc; ⑥ patients with contraindications to hormone use or shoulder hormone injections in the last 6 months; ⑦ serious osteoporosis; ⑧ serious cardiovascular/cerebrovascular/diabetes mellitus/hypertension conditions; ⑨ contraindications to the use of corticosteroids.

### 2.4. Elimination criteria

① Patients who withdrew from the study midway or were lost to follow-up; ② patients undergoing surgery after treatment were eliminated from the analysis.

**Figure 1.**
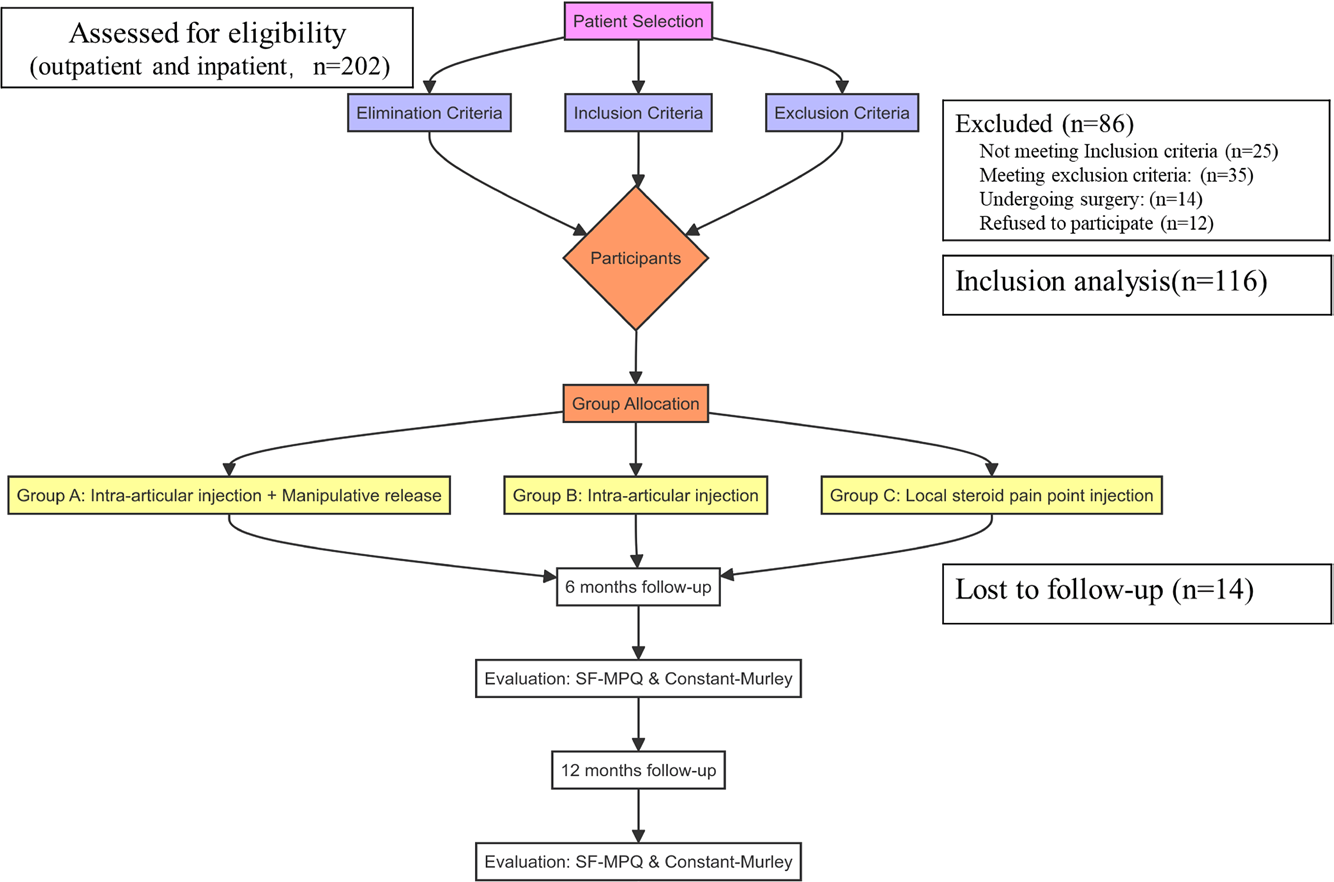
Flow chart of the study. This diagram illustrates the process of patient selection and grouping in the study, detailing the inclusion and exclusion criteria, as well as the follow-up and analysis process.

Following the implementation of the “nadir” criterion, 116 patients were included in the analysis, including the characteristics of the target population, such as age, gender, duration of the disease, and the side of the affected shoulder. Patients were allocated to 3 distinct treatment groups based on their respective treatment regimens: Group A (intra-articular injection combined with manipulative release), Group B (intra-articular injection), and Group C (local steroid pain point injection), with 40, 38, and 38 cases included, respectively. Informed consent was obtained from all patients before treatment. See Figure 1 for a flow chart.

### 2.5. Treatment method

#### 2.5.1. Local steroid pain point injection^[6]^

Patients were positioned in a sitting position, and the affected shoulder was exposed for local routine iodine povidone disinfection. Acupressure was applied to select 2 to 3 pain points, typically including the rostral prominence, supraspinatus tendon, under the acromion, and the long head tendon of the biceps brachii. Lidocaine 100 mg, betamethasone 5 mg, and saline 5 mL were used for local closure treatment. The injection depth was approximately 2 to 5 cm.

#### 2.5.2. Intra-articular cavity injection^[10]^

**Figure 2.**
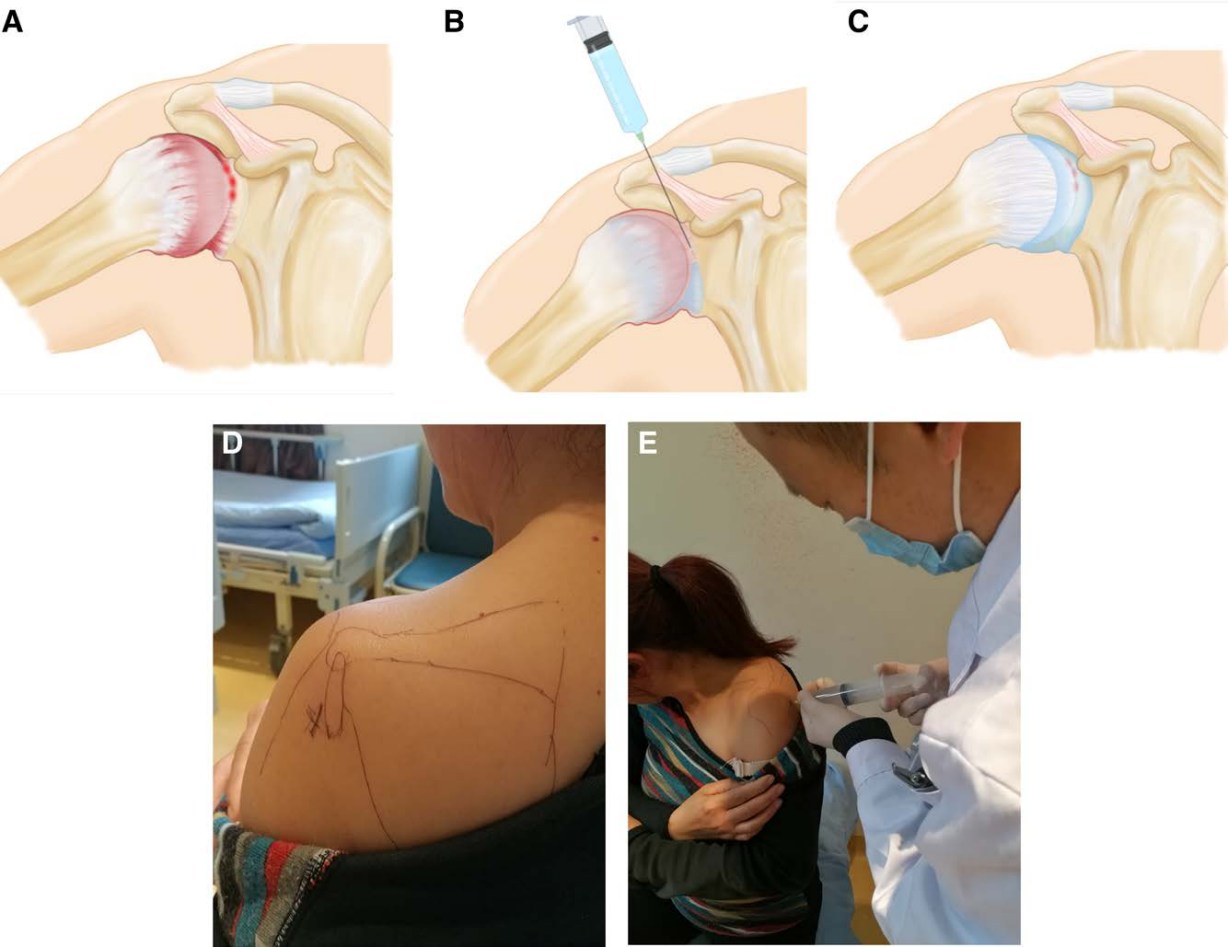
Injection procedure schematic. (A) Simulation before treatment: shows thickening of the joint capsule, congestion of the synovium, and severe adhesion. (B) Simulation during treatment: hydraulic diffusion and expansion treatment, with the puncture needle passing through the joint capsule and entering the joint cavity to pressurize and infuse medication. (C) Simulation after treatment: reduction of joint capsule adhesion and increased joint capsule volume after hydraulic diffusion and expansion treatment. (D) Surface positioning: depicts the external positioning and alignment of the patient during the injection process. (E) Injection operation: demonstrates the step-by-step technique used during the joint cavity injection.

Patients were positioned in a sitting position, with the affected side of the upper limb abducted at 90° with internal retraction. The area was disinfected, including the rostral prominence and the humeral tuberosity, which served as the point of entry for the needle. The needle and the point of entry were angled at 45° to 60°, and the needle punctured the skin, subcutaneous tissues, deltoid muscle, deltoid muscle subacromial capsule, and supraspinatus muscle. The needle then passed through the joint capsule and entered the joint cavity to a depth of approximately 6 to 8 cm. After confirming the absence of blood, the drug solution in the syringe (lidocaine 100 mg, betamethasone 5 mg, and saline 100 mL) was injected into the joint cavity at a uniform speed. Due to the narrowness of the frozen shoulder joint capsule, there was some resistance during the injection, which increased as the amount of the drug increased. The injection schematic diagram is shown in Figure 2.

#### 2.5.3. Manipulative release technique

The patient is positioned in a seated position and given time for the local anesthetic to take effect, ensuring relief from shoulder pain. The operator stands behind the affected side of the patient, using one hand to stabilize the shoulder joint while gripping the proximal end of the affected upper arm with the other hand. Through gentle maneuvers, such as internal rotation, external rotation, abduction, and adduction, the operator performs passive movements to release the adhesions. Each direction is repeated approximately 15 times.

Next, the shoulder is raised to its maximum abduction, allowing the surgeon to palpate the tension caused by adhesions. An upward force is then applied to the affected arm, usually resulting in 1 to 3 tearing sounds. It is important to note that during this procedure, some patients may experience pain and exhibit movement. In such cases, the operator may temporarily restrain the patient using their thighs, or an assistant can apply pressure to the patient’s hip with both hands to immobilize them. Caution must be exercised to avoid excessive force that may lead to fractures.

**Figure 3.**
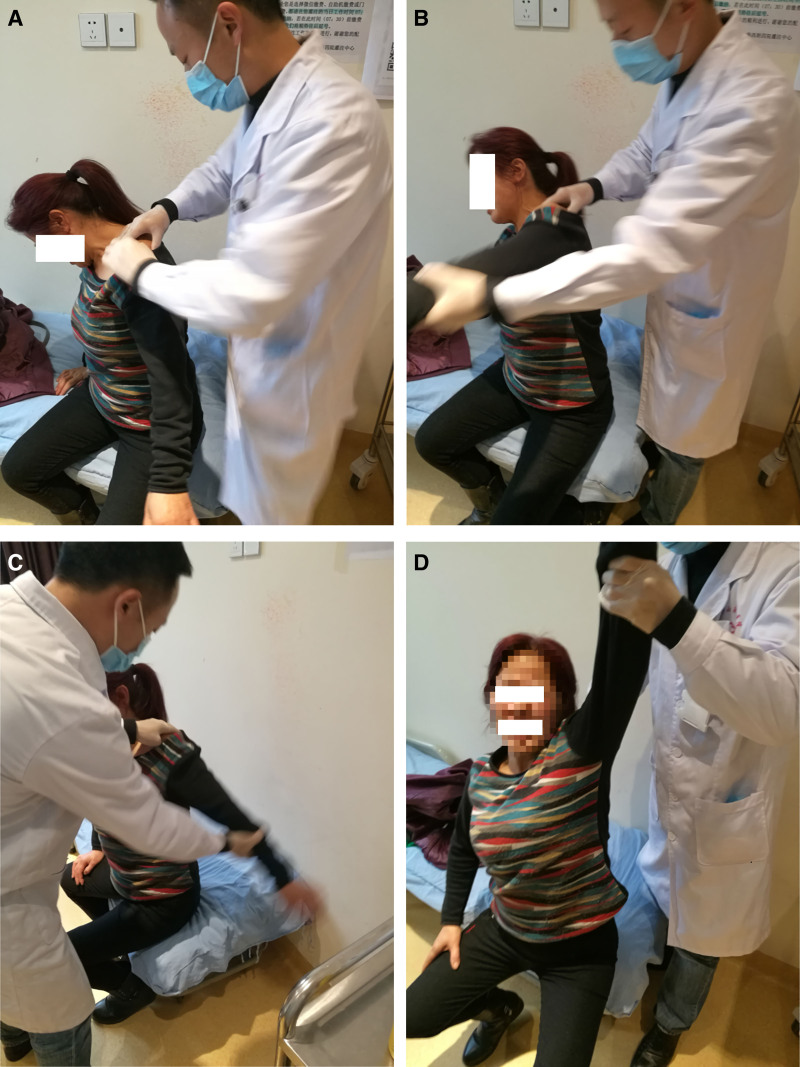
Manual muscle release procedure. (A) Manual muscle release after intra-articular injection. (B) Forward shoulder shake repeated 15 times. (C) Back shoulder shake repeated 15 times. (D) Passively abduct the arm to its end range while applying gentle outward (lateral) traction, highlighting the points of resistance and release.

**Figure 4.**
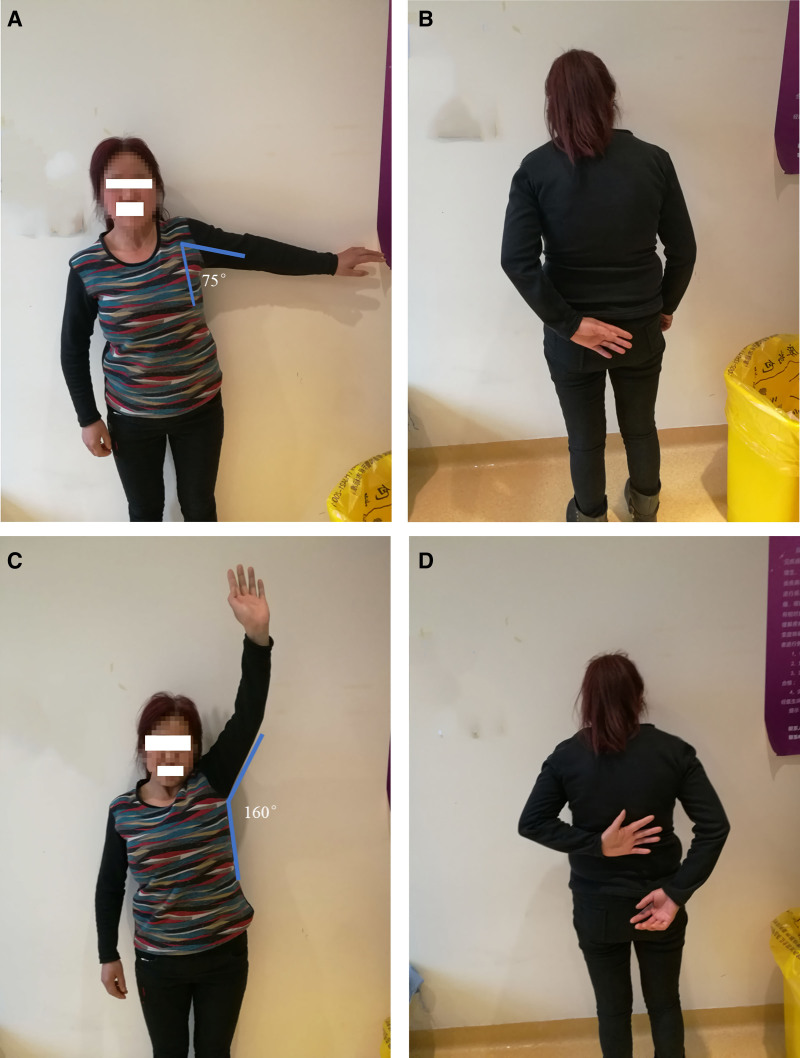
Range of shoulder joint movement before and after treatment. (A) Limited left shoulder movement before treatment with approximately 75° of abduction. (B) Posterior extension of the left hand at the level of the sacrococcygeal vertebra before treatment. (C) Improved left shoulder abduction to approximately 160° immediately after treatment. (D) Improved extension at the level of the thoracic 12th vertebra immediately after treatment.

For a visual representation of the procedure, please refer to Figure 3 in the operation schematic. Additionally, Figure 4 demonstrates the range of shoulder joint movement before and after the patient received intra-articular injection combined with manipulative release treatment.

### 2.6. Clinical efficacy evaluation

The clinical efficacy of the intervention was assessed through a rigorous evaluation process at 6- and 12-month follow-up time points. These specific follow-up durations were selected to comprehensively evaluate the stability of the treatment response in our orthopedic study. To assess the patient’s pain and function, we utilized well-established evaluation tools, namely the SF-MPQ and the CMS. The CMS encompassed 4 key dimensions, including pain, activities of daily living, ROM, and muscle strength, providing a comprehensive evaluation of shoulder joint function. For our efficacy analysis, we employed stringent criteria to determine treatment outcomes. Patients who experienced complete disappearance of shoulder pain, restoration of normal or near-normal shoulder joint activities, and the ability to resume normal life and work were classified as “cured.” Those who exhibited a significant reduction in shoulder pain and complete restoration of shoulder joint function were categorized as having an “obvious effect.” Patients who demonstrated reduced shoulder pain and improvements in functional activities were deemed “effective.” Conversely, patients who exhibited no improvement in symptoms after treatment were classified as “ineffective.” Follow-up visits consisted of a combination of outpatient appointments and telephone follow-up, constrained by the geographical distance between the patients and the hospital.

### 2.7. Statistical methods

Statistical analysis was performed using SPSS 20.0 software (IBM Corp., Armonk, NY) to evaluate the data. Descriptive statistics, including mean and standard deviation, were used to represent the central tendency and dispersion of normally distributed data. Group comparisons were conducted using appropriate statistical tests. Continuous variables were analyzed by analysis of variance (ANOVA) with multiple sample sizes. Categorical data were analyzed using the chi-square test or Fisher exact probability test, as appropriate. Statistical significance was set at a *P*-value <.05, indicating a significant difference between groups. All statistical tests were two-tailed.

### 2.8. Literature systematic review

A systematic literature review was conducted by searching PubMed from January 2004 to February 2024, encompassing the past 20 years of relevant literature available in the database. The search aimed to identify randomized controlled trials written in English that compared the outcomes of corticosteroid hydrodilator injections for frozen shoulder. The inclusion criteria for the selected studies encompassed information on the administration of cortisol, type and dosage of anesthetic, utilization of contrast agents, the volume of saline injected, injection site, number of patients, the method employed for puncture guidance, observations of outcomes, and follow-up time points.

## 3. Results

### 3.1. Follow-up statistics of the 3 groups

Among the 116 patients, 14 patients did not complete the full follow-up (3 patients in Group A, 5 patients in Group B, and 6 patients in Group C), and the remaining 102 patients completed the treatment and the full follow-up (37 patients in Group A, 33 patients in Group B, and 32 patients in Group C). There was no statistically significant difference in the age, sex, duration of the disease, and side of the affected shoulder of the patients in the 3 groups (*P* > .05, refer to Table 1).

**Table 1:** Demographic and clinical characteristics of patients in the 3 groups.

	A group	B group	C group
Age (years)	53.96 ± 5.29*	52.53 ± 5.48	53.21 ± 5.26
Sex (male/female)	7/30	5/28	5/27
Duration (months)	3.52 ± 1.64†	3.23 ± 1.36	3.58 ± 1.43
Affected side (left/right)	19/18	15/18	17/15

* *P* = .68, compared with Group B and Group C.

† *P* = .28, compared with Group B and Group C.

Comparison of SF-MPQ pain scores and CMSs among the 3 groups before treatment showed no statistically significant differences (*P* > .05). However, after treatment, there was a significant decrease in SF-MPQ pain scores and a significant improvement in CMSs at both the 6-month and 12-month follow-up time points (*P* < .001). Group A demonstrated superior results compared to Groups B and C in terms of SF-MPQ pain scores and CMSs at both follow-up time points, with statistically significant differences (*P* < .001, refer to Table 2). At the 12-month follow-up, the total effective rate was 94.6% in Group A, 90.9% in Group B, and 87.5% in Group C, with no statistically significant difference observed (*P* > .05, refer to Table 3). No serious complications were reported in any of the 3 groups.

**Table 2:** Comparison of SF-MPQ pain scores and Constant–Murley scores before and after treatment among the 3 groups.

	Group A	Group B	Group C	*P* (time effect)	*P* (groups)
SF-MPQ score				<.001	
Baseline	38.45 ± 2.24	37.52 ± 2.16	36.23 ± 2.78		.699
6 months	12.43 ± 1.63	18.46 ± 1.96	20.54 ± 1.97		<.001
12 months	10.43 ± 1.14	16.43 ± 1.65	19.54 ± 1.67		<.001
Constant–Murley score				<.001	
Baseline	44.40 ± 2.12	43.43 ± 2.14	44.26 ± 2.72		.442
6 months	89.45 ± 3.15	79.47 ± 3.45	74.16 ± 3.22		<.001
12 months	94.45 ± 3.33	84.36 ± 3.46	79.35 ± 3.64		<.001

All baseline assessments (SF-MPQ scores, Constant–Murley scores) were not significantly different between the 3 groups (*P *≥ .05). Values are expressed as mean ± SD.

Group A: intra-articular injection combined with manipulative release, Group B: intra-articular injection, and Group C: local steroid pain point injection. Repeated-measures ANOVA (Kolmogorov–Smirnov).

**Table 3:** Comparison of efficacy among the 3 groups after 12 months of treatment.

Group	Curative effect				Total effective rate
	Cure	Significant	Effective	Ineffective	
A group	20	10	5	2	94.6%*
B group	14	9	7	3	90.9%
C group	11	12	5	4	87.5%

* Based on the results of Fisher exact test, *P* = .65.

### 3.2. Literature review summary

A total of 14 papers met the inclusion criteria established for this study. Triamcinolone acetonide was the most commonly used corticosteroid, with doses typically ranging from 40 to 80 mg. Lidocaine was the predominant choice for anesthetic administration (9/14), with volumes ranging from 1 to 6 mL. Bupivacaine (3/14), ropivacaine (3/14), and concurrent use of both anesthetics in 3 cases (3/14) were also reported, while 1 study utilized sodium vitrate injections. Saline volumes varied between 10 and 40 mL, resulting in a total injection volume of 14 to 50 mL. Contrast media were employed in 2 out of 14 papers (2/14). Posterior puncture sites were the most commonly selected (8/14), followed by anterior punctures (3/14). One study directly compared anterior and posterior punctures (1/14), while the authors of 2 papers did not describe the puncture points. Ultrasound guidance was the primary method employed for puncture guidance (12/14), with fluoroscopic guidance being less frequently employed (2/14). The shortest follow-up period reported was 1 week, while the longest follow-up period was 2 years. The most frequently utilized outcome measure was the SPADI (Shoulder Pain and Disability Index). See table 4 for details.

**Table 4:** Description and summary of the literature review.

Year	Authors	Country/region	Cortisol	Anesthetic	Contrast medium	Saline	Total injection volume	Injection site	Patients number		Evaluation indicators	Follow-up time	Real-time guidance
2008^[11]^	Tveitå EK et al	Norway	Triamcinolone 2 mL	Bupivacaine 3–4 mL	Lopromide 3–4 mL	10 mL	21 mL	Glenohumeral joint (anterior)	39	SPADI		6 week	X-ray
2016^[10]^	Yoon JP et al	South Korea	Triamcinolone 40 mg/mL 1 mL	2% Lidocaine 4 mL	NA	40 mL	45 mL	Glenohumeral joint (anterior)	28	VAS, SST, Constant score		1, 3, and 6 month	X-ray
2017^[12]^	Lee DH et al	Republic of Korea	Triamcinolone 40 mg/mL 1 mL	1% Lidocaine 6 mL	NA	0.25 mL/s	25.1 ± 6.1 mL	Glenohumeral joint (posterior)	32	SPADI, VAS		3, 6, and 12 week	Ultrasound-guided
2018^[13]^	Gallacher S et al	UK	Triamcinolone 80 mg 1 mL	2% Lidocaine 4 mL	Contrast medium	40 mL	45 + mL	Glenohumeral joint (anterior)	25	OSS, EQ-5D VAS， ROM		6 week, 3 month	Ultrasound-guided
2019^[14]^	Paruthikunnan SM et al	India	Methylprednisolone 80 mg 2 mL	0.25%Bupivacaine 12 to 18 mL	NA	NA	14–20 mL	Glenohumeral joint (posterior)	44	SPADI, VAS		2 year	Ultrasound-guided
2020^[15]^	Elnady B et al	Egypt	Prednisolone 40 mg 1 mL	2% Lidocaine 1 mL	NA	15 mL	17 mL	Glenohumeral joint (anterior/ posterior)	60	VAS, SPADI, ROM		3 month	Ultrasound-guided
2020^[16]^	Yang CY et al	China, Taiwan	Triamcinolone 10 mg/mL 4 mL	2% Lidocaine 4 mL	NA	12 mL	20 mL	Glenohumeral joint (posterior lateral-medial)	53	SPADI, VAS		2 month	Ultrasound-guided
2022^[17]^	Albana R et al	Indonesia	Triamcinolone 40 mg	0.5%Bupivacaine 4 mL, 2%Lidocaine 4 mL	NA	15–20 mL	20 ± mL	Glenohumeral joint (posterior)	31	VAS, ASES, DASH		1, 6 month	Ultrasound-guided
2022^[18]^	Dai Z et al	China	Triamcinolone 50 mg	Ropivacaine 100 mg	NA	10–20 mL	20 mL	Glenohumeral joint (posterior), subacromial space	72	UCLA, DASH, ROM		1, 4, 12, 24 week, and 1 year	Ultrasound-guided
2023^[19]^	Swaroop S et al	India	Triamcinolone 40 mg/mL 2 mL	2%Lignocaine 4 mL, 0.2%Ropivacaine 4 mL	NA	40 mL	50 mL	Glenohumeral joint (posterior)	54	SPADI		2, 6 week and 3 month	Ultrasound-guided
2023^[20]^	Wang JC et al	China, Taiwan	Triamcinolone 10–40 mg	2% Lidocaine 4 mL	NA	12–15 mL	20 mL	Glenohumeral joint (posterior)	84	VAS, SPADI, ROM		6, 12 week	Ultrasound-guided
2023^[21]^	Flintoft M et al	UK	Triamcinolone 40 mg	Local anesthetic 10–25 mL	NA	NA	20–25 mL	Glenohumeral joint	55	OSS		9 month	Ultrasound-guided
2024^[22]^	Pimenta M et al	Portugal	Betamethasone 40 mg/mL 1 mL	1% Lidocaine 3 mL, 0.25%Ropivacaine 3 mL	NA	≤40 mL	≤47 mL	Glenohumeral joint (posterior)	149	DASH, VAS		1, 3, and 6 month	Ultrasound-guided
2024^[23]^	Wu SY et al	China, Taiwan	NA	NA	NA	17.5 mL	20 mL (hyaluronic acid + saline)	Glenohumeral joint	31	Constant score, SPADI, NRS, SF-36		6, 12 week	Ultrasound-guided

ASES = American Shoulder and Elbow Surgeons, DASH = disabilities of the arm, shoulder, and hand, EQ-5D = EuroQoL-5D, NRS = Numerical Rating Scale, OSS = Oxford Shoulder Score, ROM = range of motion, SDQ = Shoulder Disability Questionnaire, SF-36 = 36-item Short-Form Health Survey, SPADI = Shoulder Pain and Disability Index, SST = Simple Shoulder Test, UCLA = University of California, Los Angeles Shoulder Score, VAS = Visual Analog Scale.

## 4. Discussion

Frozen shoulder can be classified into secondary and primary categories based on its etiology. Secondary frozen shoulder can be caused by shoulder trauma, such as prolonged braking in proximal humerus fractures, or it can be caused by systemic diseases, such as Parkinson disease, which leads to decreased mobility of the trunk and limbs, resulting in limb stiffness and reduced shoulder joint movement. This, in turn, leads to chronic aseptic inflammation of the bursa, tendons, muscles, and ligaments, causing shoulder pain and decreased ROM.^[11,12]^ Additionally, hyperlipidemia is recognized as a risk factor for primary frozen shoulder, and the prevalence of frozen shoulder is 5 times higher in patients with diabetes compared to those without diabetes.^[13,14]^

Various treatment modalities have been employed to alleviate the symptoms of frozen shoulder, including functional exercises, oral medications, localized steroid injections for pain relief, platelet-rich plasma injections, as well as acupuncture and physical therapy.^[15–18]^ However, patients often struggle to adhere to shoulder joint exercises due to pain, and the pain is difficult to alleviate due to limited joint ROM. Pure oral drug treatments can provide pain relief but may not effectively improve joint ROM. Although acupuncture has shown some efficacy in treating frozen shoulder, its effectiveness is operator-dependent, and the learning curve for acupuncture is steep, limiting its widespread use. In recent years, shoulder arthroscopic intracapsular release has shown promising clinical outcomes in the treatment of refractory frozen shoulder. However, this procedure is technically demanding and requires expensive equipment, making it impractical for widespread implementation in primary hospitals. Ultrasound-guided pulsed radiofrequency has also been evaluated for the treatment of frozen shoulder, showing pain relief and improved functional limitations after 12 weeks of treatment. However, further validation is needed due to the short follow-up period.^[19,20]^

Therefore, our team is trying to find safe, effective, easy, and inexpensive equipment, and no special equipment is needed to treat frozen shoulder. In our study, we observed that the utilization of intra-articular injection combined with manipulative release demonstrated superior efficacy as a treatment modality for frozen shoulder. In our study, we developed a treatment protocol that is safe, effective, easy to perform, and cost-effective. Although previous studies have utilized a combination of steroid and water dilation for frozen shoulder treatment,^[21–25]^ the injection volumes reported in those studies were comparatively smaller than the present study. In our literature review study, the injection volume ranged from 14 to 50 mL, while in our study, we utilized 100 mL of saline. It is important to note that during the treatment process, we observed that many patients did not experience any noteworthy discomfort, except for a temporary sensation of distension and pain in the affected shoulder. Furthermore, a higher percentage of patients demonstrated significant effectiveness in terms of pain relief and improved ROM immediately after receiving injections with larger doses of saline. However, it is important to note that our study did not specifically focus on the occurrence of shoulder capsule rupture as a result of the extensive hydrodilation procedure. We administered a drug solution (lidocaine 100 mg + betamethasone 5 mg + saline 100 mL) into the joint cavity under pressure, utilizing the analgesic effect of lidocaine, the anti-inflammatory effect of betamethasone to reduce aseptic inflammation, and the hydraulic effect of the large volume saline injection to diffuse the expansion of the adhesive joint capsule, effectively improving joint function. This protocol has demonstrated no serious adverse reactions, with only a few cases of tachycardia, dizziness, and nausea during the puncture procedure, which can be alleviated by pausing the operation, lying down and resting, and providing oral glucose solution. Although ultrasound-guided injections are commonly perceived as safer than non-ultrasound-guided injections, previous studies have shown no statistically significant differences in terms of injection accuracy, pain relief, improvement in joint mobility (ROM), and functional scores.^[8]^ Moreover, it is important to consider that ultrasound-guided injections are associated with higher costs and longer procedure times. Additionally, interventional punctures under X-ray fluoroscopy pose a potential radiation risk to both the patient and the operator. In our study, we mitigated the need for interventional guidance by ensuring precise positioning of the needle prior to puncture and observing fluid reflux at the needle tip, thereby eliminating the dependence on interventional guidance during the procedure. Thus, intra-articular injections combined with manipulative release for the treatment of frozen shoulder fulfill the requirements of being safe, effective, easy to perform, and cost-effective.

In our initial attempts, performing manipulative release during the operation process resulted in a higher failure rate and no pain relief. Even if the release was successful, patients often experienced a worsening of symptoms. Some researchers performed manipulative release under intravenous anesthesia, which improved the success rate of capsular adhesion release. However, patients often experienced severe pain in the affected shoulder after awakening from anesthesia, leading to reluctance to perform further functional exercises and recurrence of capsular adhesion. In our protocol, we performed manipulative release after injection treatment in the joint cavity. The hydraulic pressure and analgesic effect of the drug made the procedure tolerable for patients, resulting in a higher success rate of release. Some patients even experienced immediate significant improvement in shoulder joint ROM after the completion of drug infusion, despite the absence of manipulative release.

## 5. Limitations

This study has several limitations that warrant consideration. First, its retrospective design may have introduced confounding factors, limiting our ability to establish causal inferences. Additionally, the 12-month follow-up period was relatively short; extending this period would have provided a more comprehensive understanding of the long-term impact and sustainability of the treatment effect, which we aim to explore in future studies. Furthermore, we faced challenges with traditional outpatient follow-up, necessitating telephone follow-ups in some instances. During these calls, we assessed the CMS by guiding patients through self-assessments of pain, daily activities, perceived ROM, and inferred muscle strength based on reported functional capacity. While this method may introduce subjectivity and potentially incomplete or unreliable scores, it was necessary under the circumstances. We adapted our analysis by using weighted means based on the type of follow-up to address these potential discrepancies. Future studies should prioritize in-person follow-ups whenever possible to mitigate this limitation. Moreover, some patients used additional oral and topical nonsteroidal analgesics and herbal patches during follow-up, which may have introduced confounding factors that could bias the results. Due to sample size limitations, it was not feasible to completely exclude these cases. However, future studies could address this issue by expanding the sample size and implementing stricter inclusion and exclusion criteria. Lastly, it is noteworthy that no specific arthrography or musculoskeletal ultrasound guidance was conducted before or after the injection in this study. This limitation hindered our ability to identify adhesion points and objectively assess the extent of joint capsule expansion before and after capsular dilation. Therefore, operators should have experience with ultrasound-guided punctures before undertaking this treatment protocol.

## 6. Conclusion

In conclusion, our study demonstrates that the combination of intra-articular injection and manipulative therapy provides rapid pain relief and substantial improvement in joint function for patients with frozen shoulder. This treatment approach is safe, does not necessitate specialized tools, has a short learning curve, and is suitable for implementation in hospitals of all levels.

## Acknowledgments

We would like to express our gratitude to the radiologists in our hospital for their valuable contribution, as well as to Dr Dong-Xin Huang for his expertise in creating the schematic diagrams.

## Author contributions

**Data curation:** Chengwei Kang, Hong-Yun Wo, Li-Xue Wu.

**Methodology:** Li-Xue Wu, Zhao-Kui Yan.

**Writing – original draft:** Chengwei Kang.

**Writing – review & editing:** Zhao-Kui Yan.

## Supplementary Material
