# Supplementary material for: A retrospective study on the short-term efficacy of injection and manipulation in primary frozen shoulder
Source: Medicine (Baltimore). 2025 May 16;104(20):e42310. doi: 10.1097/MD.0000000000042310 (PMC12091637; doi:10.1097/MD.0000000000042310)
Supplement: Supplementary file 1 [file medi-104-e42310-s001.docx]

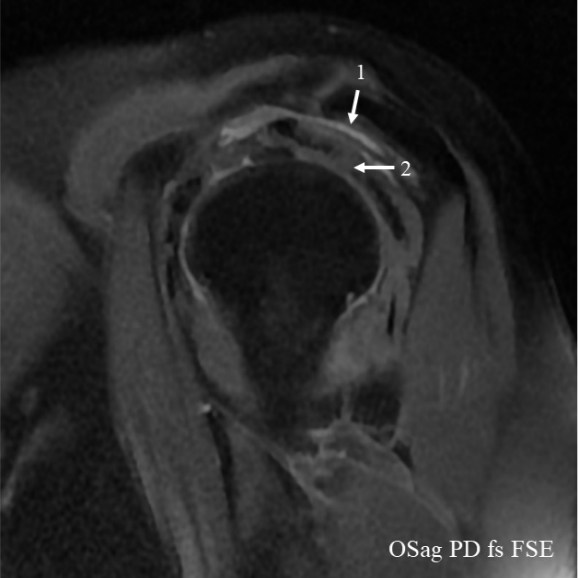

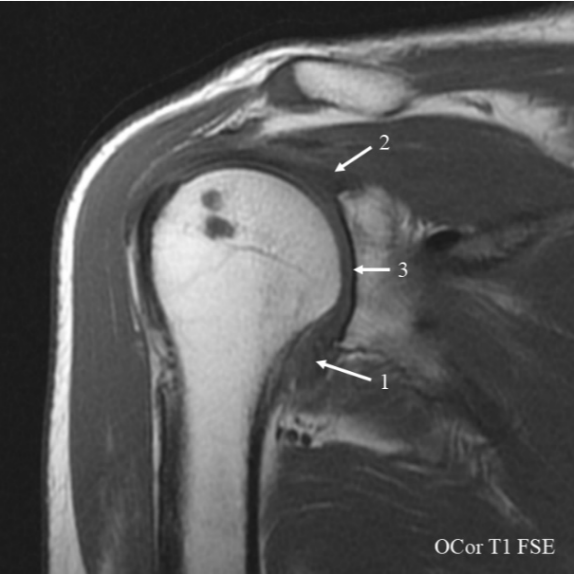


Fig.S1 (a) Fig.S1 (b)


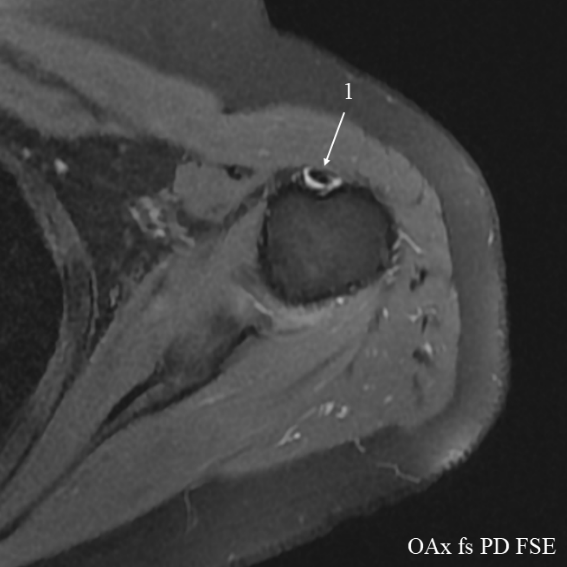

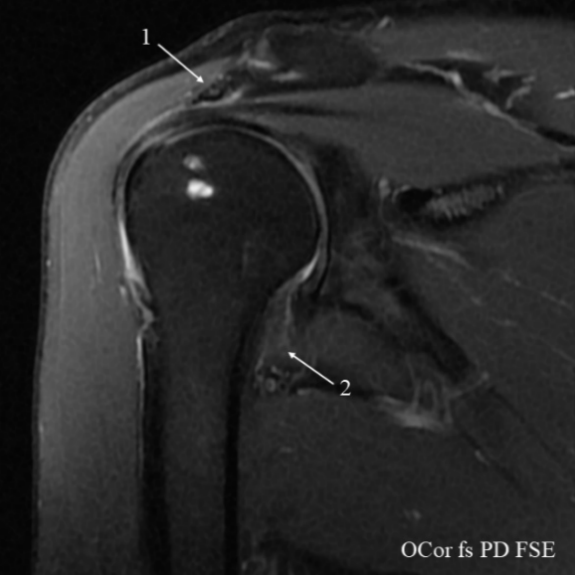


Fig.S1 (c) Fig.S1 (d)

**Supplementary Fig. S1** The magnetic resonance image (MRI) findings in a 53-year-old woman with left frozen shoulder are presented in Fig. S1. The image highlights the following observations: (a) Subacromial synovitis is depicted by 1, while 2 indicates supraspinatus tendinitis (arrows). (b) The MRI reveals nodular thickening and adhesion of the inferior glenohumeral ligament, partial occlusion of the subcoracoid adipose triangle, and thickening of the joint capsule wall, denoted by 1, 2, and 3, respectively (arrows). (c) Biceps long tendon tendinitis is indicated by 1 (arrow). (d) The image shows rostro-humeral ligamentitis represented by 1, alongside thickening of the inferior glenohumeral ligament indicated by 2 (arrows).
